# Modeling of Retina and Optic Nerve Ischemia–Reperfusion Injury through Hypoxia–Reoxygenation in Human Induced Pluripotent Stem Cell-Derived Retinal Ganglion Cells

**DOI:** 10.3390/cells13020130

**Published:** 2024-01-11

**Authors:** Tomoyo Yoshida, Tadashi Yokoi, Taku Tanaka, Emiko Matsuzaka, Yuki Saida, Sachiko Nishina, Shuji Takada, Shigeomi Shimizu, Noriyuki Azuma

**Affiliations:** 1 National Center for Child Health and Development, 2-10-1, O-kura, Setagaya-ku, Tokyo 1578535, Japan; uemura-t@ncchd.go.jp (T.Y.); yokoi-t@ncchd.go.jp (T.Y.); matsuzaka-e@ncchd.go.jp (E.M.); nishina-s@ncchd.go.jp (S.N.);; 2 Department of Pathological Cell Biology, Tokyo Medical and Dental University, 1-5-4, Yushima, Bunkyo-ku, Tokyo 1138510, Japan; shimizu.pcb@mri.tmd.ac.jp; 3 Department of ophthalmology, Kyorin University, 6-20-2, Arakawa, Mitaka, Tokyo 1818611, Japan; 4 Department of Developmental and Regenerative Biology, Tokyo Medical and Dental University, 1-5-4, Yushima, Bunkyo-ku, Tokyo 1138510, Japan

**Keywords:** retinal ganglion cells, ischemia–reperfusion, hypoxia–reoxygenation, human iPS cells, apoptosis

## Abstract

Retinal ganglion cells (RGCs) are specialized projection neurons that constitute part of the retina, and the death of RGCs causes various eye diseases, but the mechanism of RGC death is still unclear. Here, we induced cell death in human induced pluripotent stem cell (hiPSC)-derived RGC-rich retinal tissues using hypoxia–reoxygenation in vitro. Flow cytometry, immunochemistry, and Western blotting showed the apoptosis and necrosis of RGCs under hypoxia–reoxygenation, and they were rescued by an apoptosis inhibitor but not by a necrosis inhibitor. This revealed that the cell death induced in our model was mainly due to apoptosis. To our knowledge, this is the first model to reproduce ischemia–reperfusion in hiPSC-derived RGCs. Thus, the efficacy of apoptosis inhibitors and neuroprotective agents can be evaluated using this model, bringing us closer to clinical applications.

## 1. Introduction

The retina is located inside the eye and functions to sense and transmit light. It consists of a total of 10 layers: 9 layers of sensory retina and the outermost retinal pigment epithelium. The outermost rod–cone layer of the sensory retina receives light and converts it from light to electrical signals, which are transmitted via bipolar cells to the retinal ganglion cells (RGCs) and projected via the optic nerve to the lateral geniculate body, where the information is transmitted to the visual cortex [1,2]. The long axons of RGCs are susceptible to injury and cell death in various ocular diseases, including retinal vessel occlusion, glaucoma, diabetic retinopathy, ischemic optic neuropathy, hereditary optic neuropathy, and trauma [2,3,4,5], resulting in impaired visual field and acuity. In particular, ischemia-induced RGC degeneration is difficult to treat because it is caused by hypoxia and ischemia-induced hyponutrient damage, as well as by protein degeneration caused by the generation of free radicals upon reperfusion [6].

Ischemia-induced RGC degeneration involves multiple complex cell death pathways [7]. It has been reported that proteins involved in necroptosis (Ripk1 and Ripk3) are present in the retina and that the suppression of these proteins prevents necrosis caused by ischemia–reperfusion and increases RGC survival [8]. Also, the fact that retinal ischemia causes pyroptosis in RGCs via the thioredoxin-interacting protein/Nod-like receptor family pyrin domain-containing 3 has been reported [9]. Oxytosis/ferroptosis and parthanatos are also reported to be more active 24 h after I/R [10]. Autophagy also results in retinal degeneration [11] but has been reported to rescue RGCs from cellular ischemic damage under some conditions [12]. Apoptosis has been considered the major cell death pathway of ischemia–reperfusion for a long time [13,14]. Its development is largely mediated by the expression of Bax and the involvement of caspase cascades, and although the mechanism is gradually being elucidated, the full extent of the mechanism is still unknown. 

Human pluripotent stem cells (hPSCs), including embryonic stem cells (ESCs) and induced pluripotent stem cells (iPSCs), are convenient and useful for generating human-derived RGCs, elucidating pathophysiology, and estimating drug efficacy [15,16,17]. We have previously generated RGCs from human and mouse iPSCs [15]. In this model, RGCs were induced in a 2D model using a previously established 3D culture system [18] in a mixture of retinal primordial cells. Stable RGCs with functioning axons develop in the marginal areas of the retinal primordial tissue, wherein RGCs can only be maintained stably in a homogenous environment including other types of retinal cells which support RGC homeostasis.

Here, we established an in vitro model of ischemia/reperfusion (I/R) injury of the retina, consistent with hypoxia–reoxygenation (H/R) in various ischemic retinal and optic diseases, using human iPSC (hiPSC)-derived RGCs. We also investigated the types of cell death in this I/R cell model using flow cytometry and Western blotting. Immunohistochemistry was also performed to quantify the death of RGCs.

## 2. Materials and Methods

### 2.1. Human iPSC Culture

The culture method was modified from a previously described procedure [15]. Briefly, human iPSCs obtained from RIKEN BRC (Tsukuba, Japan) were maintained on a feeder layer of inactivated mouse embryonic fibroblasts (MEFs) in Primate ES medium (ReproCELL, Yokohama, Japan) supplemented with 10 ng/mL of recombinant human basic fibroblast growth factor (bFGF; Invitrogen, Waltham, MA, USA). For passaging, hiPSC colonies were detached using a dissociation solution (ReproCELL), broken into smaller pieces by gentle pipetting, and disseminated onto a new plate. Cell passaging was performed at a 1:3 to 1:4 split ratio.

### 2.2. Differentiation Protocol of RGC-Rich Retinal Tissues

Human iPSC-derived RGCs and retinal tissues were induced as previously described [15]. Briefly, hiPSCs were dissociated and resuspended in retinal differentiation medium (RDM). After separating the floating hiPSCs from feeder cells, they were seeded into V-bottomed, low-cell-adhesion, 96-well plates at 9000 cells/well (defined as D0). On D2, Matrigel was added, and on D12, the aggregates were transferred to low-cell-adhesion 24-well plates with RDM containing Matrigel and FBS. On D15, CHIR99021 and SAG were added to the medium. On D18, the aggregates were transferred to retinal maturation medium (RMM) and cultured without FBS. The adhesion culture was started on D24; aggregates were transferred to poly-D-lysine/laminin-coated 24-well plates in RMM medium containing FBS and 100 ng/mL of BDNF.

### 2.3. Hypoxia–Reoxygenation

After the aggregates were transferred to adhesion plates, the culture medium was changed to Neurobasal Medium (NM, Gibco, Life Technologies Corporation, Grand Island, NY, USA), containing 0.5 mM of L-Glutamine (Gibco) and 2% NeuroBlew-21 (MACS) on D26. Q-VD-Oph (10 µM; Abcam, Cambridge, UK), Necrox-5 (30 µM; Enzo, Farmingdale, NY, USA), and Z-LEHD-FMK (10 µM; Medical & Biological Laboratories, Tokyo, Japan) were added to the respective plates on D26. On D27, the plates were transferred and sealed in a container with AnaeroPack Kenki (Mitsubishi Gas Chemical, Tokyo, Japan), an oxygen absorber/carbon dioxide generator, to simulate hypoxic injury at 37 °C for 18 h. The plates were then removed from the container and incubated at 37 °C for 4 h under normal atmospheric conditions to simulate reoxygenation. The duration of hypoxia and reperfusion was deemed appropriate based on a previous report [19], and we further conducted multiple experiments in order to adjust the conditions under which both cell death and their rescue by the drug could be observed.

### 2.4. Flow Cytometry

RGCs with small parts of optic vesicles (OVs) were removed from the whole cell body, picked from the plate, added to 100 µL of PBS, and agitated; these were then centrifuged at 300× *g* for 3 min. After aspirating the supernatant, accutase (Gibco) was added to the cell precipitates and kept at 37 °C for 5 min. After pipetting 10 times, CellEvent^TM^ Caspase-3/7 Green Detection Reagent (0.5 M; Invitrogen) was added, agitated, and kept at 37 °C for 25 min in darkness. SYTOX^TM^ AADvanced^TM^ Dead Cell Stain (1M; Invitrogen) was added, agitated, and incubated at 37 °C for 10 min in darkness. Next, 400 µL of PBS was added and filtered through a round-bottom tube with a 35 µm strainer cap.

An Attune flow cytometer (BD BioSciences, San Jose, CA, USA) was used for the flow cytometry. The analysis was conducted five times with a minimum of 20,000 cells per condition.

### 2.5. Western Blot Assay

Each OV was harvested and sonicated in CelLyticM Cell Lysis Reagent (Millipore Sigma, Burlington, MA, USA), containing a protease inhibitor cocktail (Complete Mini-EDTA-free; Millipore Sigma). The protein concentrations were measured using the Pierce BCA Protein Assay (Thermo Fisher Scientific, Waltham, MA, USA) with bovine serum albumin as the standard. For immunoblotting, 3, 3.8, or 8 μg of each sample was loaded and run on 4–12% gradient SDS-PAGE gels (NuPAGE Bis-Tris Protein Gels, Thermo Fisher Scientific) with 2-(N-morpholino) ethane sulfonic acid buffer or 3-(N-morpholino) propane sulfonic acid buffer. The proteins were then electrotransferred to a polyvinylidene fluoride membrane at 100 V for 1 h. The membranes were blocked with 5% skim milk in Tris-buffered saline (Bio-Rad Laboratories, Hercules, CA, USA) containing 0.05% Tween 20. Each membrane was probed with primary antibodies against caspase-3 (1:250 dilution, Abcam) and β-actin (1:5000 dilution, Millipore Sigma). For β-actin, immunoreactivity was visualized with the secondary antibody conjugated to alkaline phosphatase and the AP Conjugate Substrate Kit (Bio-Rad Laboratories). For caspase-3, immunoreactivity was visualized with HRP-Conjugated Secondary Antibody Cocktail (1:100 dilution, Abcam). Images of the membranes were captured using an Image Quant LAS 4010 (Cytiva, Marlborough, MA, USA).

### 2.6. Immunohisotochemistry

The immunostaining of the axons was performed using whole cells fixed in a dish. Whole-cell specimens were fixed in 12% paraformaldehyde (pH 7.0) for 5 min at room temperature. After two rinses with PBS, the specimens were incubated overnight at 4 °C in a blocking buffer. The cells were incubated with an anti-beta tubulin antibody (T-5076, Sigma), followed by three washes with PBS and centrifugation for 10 min. Mouse-specific Alexa Fluor-488-conjugated antibodies (1:500, Invitrogen) were used as the secondary antibody for 40 min, followed by centrifugation at room temperature in the dark. After washing thrice with PBS for 5 min each, the samples were examined under a confocal microscope.

### 2.7. Image Processing

The density of the Western blots was quantified using ImageJ software (Ver 1.53, NIH, Bethesda, MA, USA), and a relative score was calculated compared to the control under normal oxygen conditions, obtained from three independent experiments.

The immunostained specimens were imaged using an IX71 inverted research microscope (Olympus, Tokyo, Japan), and the images were processed using ImageJ. To quantify the number of axons from the RGCs, the images were sharpened and converted into 8-bit binary images using ImageJ. The relative axon counts were statistically analyzed for each condition.

### 2.8. Statistics

All statistical analyses were performed using the EZR software (Ver 1.63, Jichii Medical University, Saitama Medical Center, Saitama, Japan). Data were compared between groups using one-way and two-way ANOVAs. Statistical significance was set at *p* < 0.01 (one-way ANOVA) and *p* < 0.05 (two-way ANOVA).

### 2.9. Data Collection

**Figure 1:**
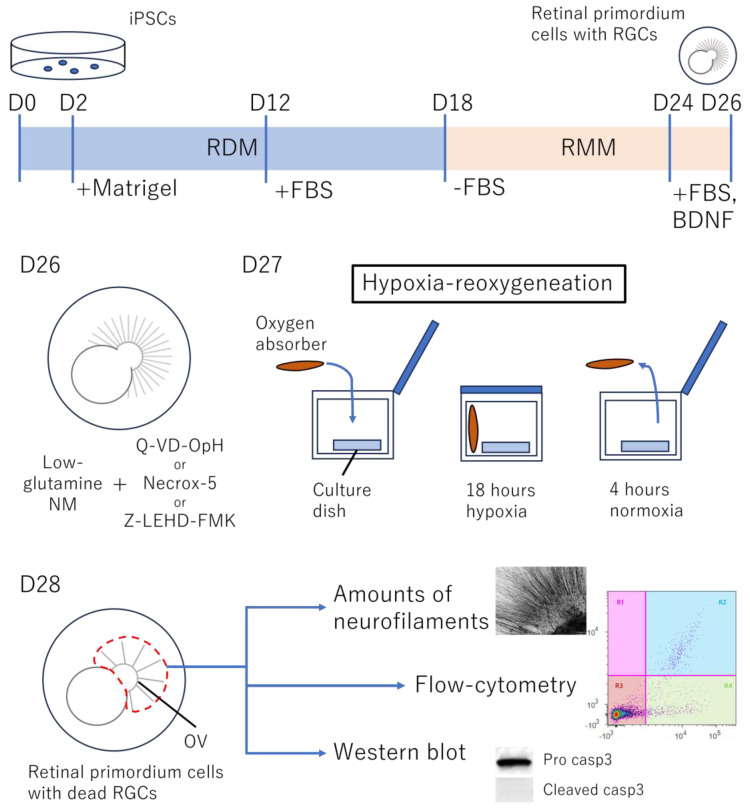
Schema of human iPSC-derived retinal ganglion cell (RGC) generation, exposure to hypoxia–reoxygenation (H/R) for cell death induction, and its analysis. The generation of human retinal primordia from human iPSCs was initiated in retinal differentiation medium (RDM) in a 3D culture system (D0) with the addition of Matrigel (D2) and FBS (D12). The floating 3D retinal primordia were further cultured with retinal maturation medium (RMM). On D24, the floating retinal primordium (embryoid body) was transferred to a 2D culture with FBS and BDNF. On D26, the culture solution was changed to low-glutamine neurobasal medium (NM). In each dish, Q-VD-Oph (10 µM), Necrox-5 (30 µM), or Z-LEHD-FMK (10 µM) was added before ischemia induction. On D27, the cells were moved and sealed in a container for exposure to hypoxia at 37 °C for 18 h, followed by incubation at 37 °C under normal air conditions for 4 h to simulate reoxygenation. On D28, the axons of RGCs elongated from the retinal primordia were observed. Small parts of the attached optic vesicle (OV), indicated by the dotted red circle, were collected and analyzed using flow cytometry, immunocytochemistry, and Western blotting. Abbreviations: RGC: retinal ganglion cell; D0, D1: day0, day1; RMM: retinal maturation medium; NM: neurobasal medium; Q-VD-Oph: pan-caspase inhibitor; Necrox-5: necrosis inhibitor; Z-LEHD-FMK: caspase-9 inhibitor; OV: optic vesicle; FBS: fetal bovine serum; BDNF: brain-derived neurotrophic factor; pro casp3: pro caspase3; cleaved casp3: cleaved caspase 3.

After inducing hiPSC-derived RGCs as reported previously, H/R and the subsequent analyses of the collected cells were performed, as shown in Figure 1.

## 3. Results

### 3.1. Induction of RGC-Rich Retinal Primordia and Cell Death by In Vitro I/R

**Figure 2:**
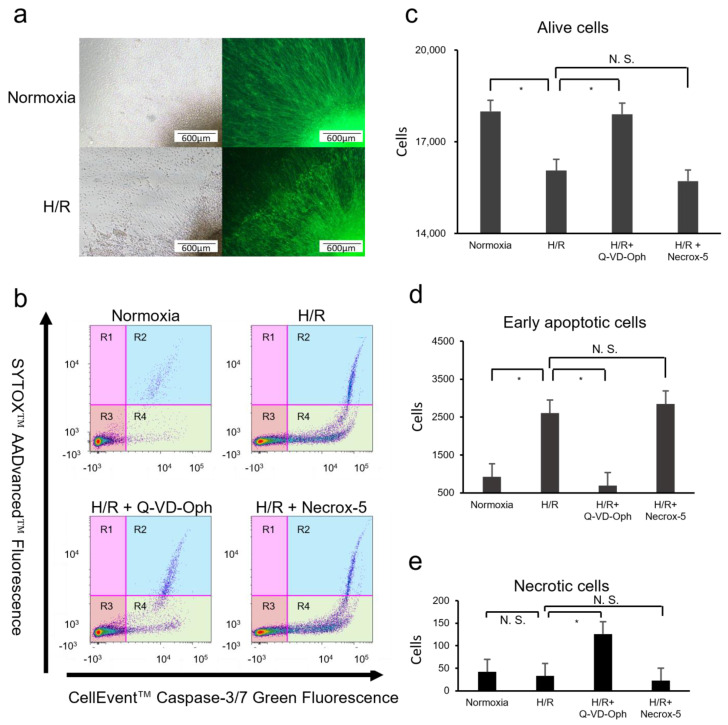
Hypoxia–reoxygenation (H/R) increased the number of apoptotic cells in human iPSC-derived RGCs compared with that in cells under normoxia. (**a**) The induction of cell death in human iPSCs-derived RGCs through exposure to in vitro ischemia and reperfusion, presented using phase contrast images and immunohistochemistry. Numerous axons from RGCs, which were located at the peripheral margin of the induced and attached retinal primordia, were identified. Disruption to RGC axons and retinal primordia was observed after H/R exposure. The axon density appeared to be reduced. The decrease in axons was further confirmed by immunohistochemistry, indicating RGC death. (**b**) Representative result of flow cytometry, analyzing apoptotic and necrotic cell death in retinal primordia under normoxia, H/R, H/R+Q-VD-Oph treatment, and H/R+Necrox-5 treatment, respectively. Using double staining with AAD and caspase3/7, the digested retinal primordium cells under each condition were analyzed using flow cytometry. The R1 region shows necrotic cells, R2 shows late apoptotic cell deaths, R3 shows live cells, and R4 shows early apoptotic cells. In cells cultured under normoxia, 95.7% of the cells were live. However, 29.8% of cells under H/R exposure showed early and late apoptosis. H/R-induced apoptosis was obviously rescued by adding the caspase inhibitor, Q-VD-Oph, whereas the necrosis inhibitor, Necrox-5, showed no effect on cell death. (**c**) Compared to the cells under normoxia, the primordium cells experienced significant cell death after H/R exposure (*p* < 0.01, two-way ANOVA), which was rescued by adding Q-VD-Oph (*p* = 0.01). No significant changes were observed in the H/R- and H/R+Necrox-5-treated cells (*p* = 0.51) (**d**) The number of early apoptotic cells in the H/R group was significantly increased compared that in the normoxia group (*p* < 0.01). Compared to the H/R group, the percentage of early apoptotic cells in the H/R+Q-VD-Oph group was significantly lower (*p* < 0.01), but no significant change was observed in the H/R+Necrox-5-treated cells (*p* = 0.63). (**e**) Necrotic cells did not differ significantly between normoxia and H/R (*p* = 0.82), nor between H/R and H/R+Necrox-5 (*p* = 0.79), but they did between H/R and H/R+Q-VD-Oph (*p* = 0.03). * *p* < 0.05. RGC, retinal ganglion cell; H/R, hypoxia–reoxygenation.

We successfully obtained RGCs with elongated axons from attaching OVs or RGC-rich retinal primordia (Figure 2a). Numerous axons from the RGCs, identified by immunostaining, were located at the peripheral margin of the induced and attached retinal primordia. After extensive H/R exposure for 22 h (18 h hypoxia and 4 h normoxia), the attached RGCs located at the periphery of the attached OVs and elongated RGC axons were disrupted.

### 3.2. Identification of the Type of Cell Death Induced by H/R Injury in hiPSC-Derived RGCs

Next, we determined the type of cell death in the H/R and control normoxic groups, focusing on apoptosis and necrosis, using flow cytometry. We analyzed apoptotic and necrotic cell death in retinal primordia under normoxia, H/R, and H/R with an apoptosis inhibitor, Q-VD-Oph, and a necrosis inhibitor, Necrox-5, respectively. A cell death assay was performed using double staining with CellEvent^TM^ Caspase-3/7 and SYTOX^TM^ AADvanced^TM^, and the digested retinal primordial cells in each condition were analyzed using flow cytometry. This assay was validated by treating the cells with staurosporine (an apoptosis inducer) and H_2_O_2_ (a necrosis inducer) (Figure S1a,b). The R1 region shows necrotic cells, R2 shows late apoptotic cell death, R3 shows live cells, and R4 shows early apoptotic cells. Of the cells cultured under normoxia, 95.7% survived. In contrast, 29.8% of the cells exposed to H/R experienced early and late apoptosis. The apoptosis induced by H/R exposure was rescued by the caspase inhibitor Q-VD-Oph, while the necrosis inhibitor Necrox-5 showed no effect on cell death (Figure 2b). Further, compared to the cells under normoxia (10.0%), 19.7% of the primordium cells experienced significant cell death after the H/R exposure (*p* < 0.01, two-way ANOVA), which was rescued by adding Q-VD-Oph (10.5%, *p* = 0.01). No significant changes were observed in the H/R- and H/R+Necrox-5-treated cells (21.4%, *p* = 0.51) (Figure 2c). The early apoptotic cells in the H/R group constituted 13.0%, significantly more compared to 4.6% in the normoxia group (*p* < 0.01). Compared to the H/R group, the percentage of early apoptotic cells in the H/R+Q-VD-Oph group was 3.4%, significantly lower (*p* < 0.01), but no significant change was observed in the H/R+Necrox-5 treated cells (14.2%, *p* = 0.63) (Figure 2d). The necrotic cells did not differ significantly between normoxia and H/R (0.2% under normoxia and 0.2% in H/R (*p* = 0.82)), nor between H/R and H/R+Necrox-5 (0.1%, *p* = 0.79), but they did between H/R and H/R+Q-VD-Oph (0.6%, *p* = 0.03) (Figure 2e).

### 3.3. RGC Death Confirmed by Western Blot and Immunohistochemistry of Axons

**Figure 3:**
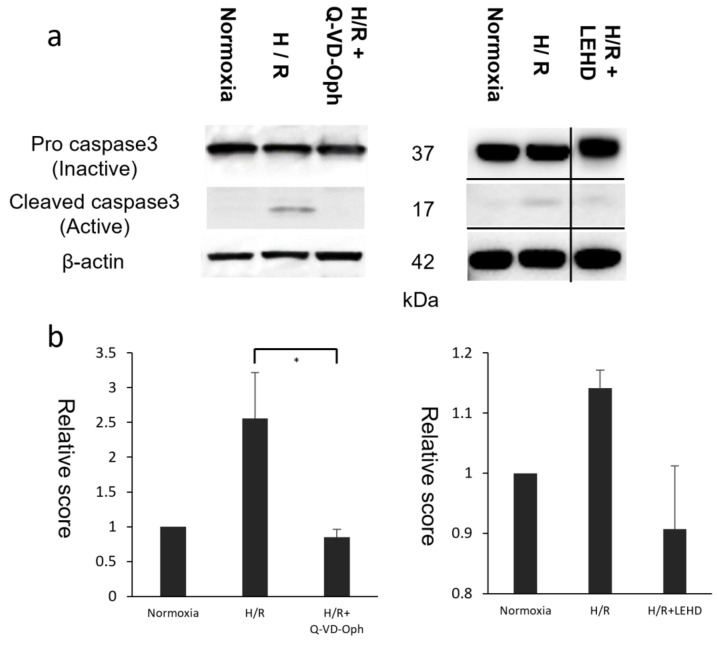
Western blot proved a decrease in the axons of RGCs under hypoxia–reoxygenation (H/R). (**a**) Western blotting for caspase3 revealed that 37kDa pro-caspase3 was cleaved to 17kDa activated caspase3 in RGCs after H/R. Pro-caspase3 cleavage was completely inhibited by Q-VD-Oph, a pan-caspase inhibitor. Further, pro-caspase3 cleavage was markedly decreased by Z-LEHD-FMK, a caspase9-specific inhibitor. (**b**) Relative scores of protein concentration compared to the normoxia group were calculated by averaging the results obtained from three independent experiments. The significant differences were between the H/R and Q-VD-Oph groups (*p* = 0.01) in terms of protein concentrations. Despite the absence of statistically significant differences, the experiments demonstrated a trend toward increased caspase3 expression for H/R over normoxia (*p* = 0.02, 0.19) and H/R over LEHD (*p* = 0.02). * *p* < 0.01. RGC, retinal ganglion cell; H/R, hypoxia–reoxygenation; Pro casp3, pro caspase3; cleaved casp3, cleaved caspase3.

The suppression of the H/R effect upon Q-VD-Oph administration was also confirmed by the Western blot analysis (Figure 3a). The Western blotting for caspase3 revealed that 37kDa pro-caspase3 was cleaved to the 17kDa activated caspase3 in the RGCs after H/R, whereas pro-caspase3 cleavage was completely inhibited by Q-VD-Oph. Further, pro-caspase3 cleavage was markedly decreased by Z-LEHD-FMK, a caspase9-specific inhibitor. The relative scores of protein concentration compared to the normoxia group were calculated by averaging the results obtained from three independent experiments (Figure 3b). The significant differences were between the H/R and Q-VD-Oph groups (*p* = 0.02) and between the H/R and LEHD groups (*p* = 0.04) in terms of protein concentrations. Despite the absence of statistically significant differences (*p* = 0.06), the experiments demonstrated a trend toward increased caspase3 expression for H/R over normoxia.

**Figure 4:**
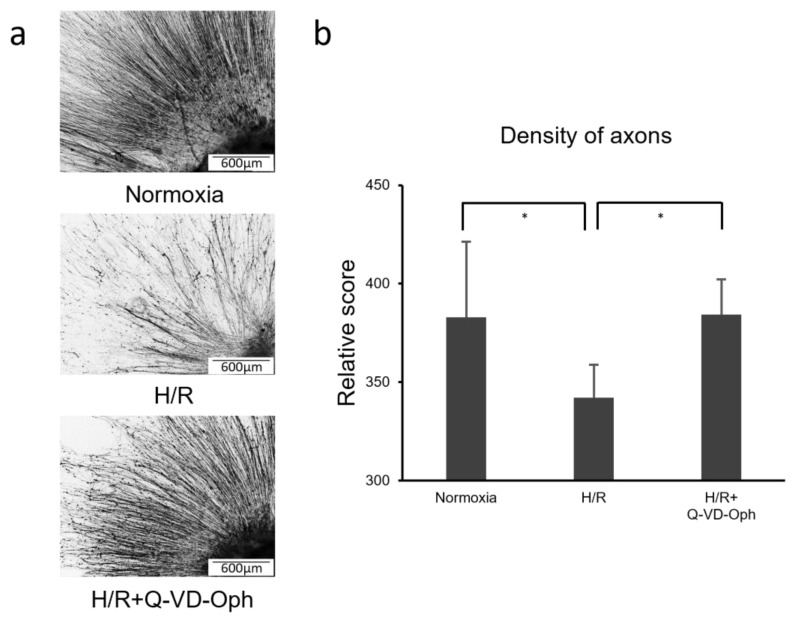
Immunofluorescence visually proved a decrease in the number of axons of retinal ganglion cells (RGCs) under hypoxia–reoxygenation (H/R). (**a**) Images of the optic vesicles (OV)s and axons under normoxia, H/R, and H/R+Q-VD-Oph after immunostaining were obtained and converted to binary form using Image J. (**b**) Comparison of the mean fluorescence-stained area in the image, excluding the OVs, showed that the area was significantly lower for the H/R group than for the normoxia group (*p* < 0.01, one-way ANOVA). However, there was no significant difference between the normoxia and H/R+Q-VD-Oph treatments (*p* = 1.0). * *p* < 0.01. RGC, retinal ganglion cell; H/R, hypoxia–reoxygenation; Pro casp3, pro caspase3; cleaved casp3, cleaved caspase3; OV, optic vesicle.

We further quantified the number of RGC axons by immunohistochemistry. The cells in the normoxia, H/R, and H/R+Q-VD-Oph groups were fixed, immunostained, and binary converted using ImageJ (Figure 4a). The normoxia- and H/R+Q-VD-Oph-treated cells showed more axons than the H/R-treated cells. A comparison of the mean relative counts of stained axons in the image area, excluding the OVs, showed significantly lower counts in the H/R-treated cells than in the normoxic cells (*p* < 0.01, one-way ANOVA) (Figure 4b). In contrast, there was no significant difference between the normoxia- and H/R+Q-VD-Oph-treated cells (*p* = 1.0), indicating that apoptosis in RGCs was rescued by Q-VD-Oph.

## 4. Discussion

We created an H/R model that reproduces ischemia–reperfusion injury using hiPSCs. To induce human RGC death in vitro, we used an H/R model, which involves ischemia and reoxygenation under glucose-deprived culture conditions, as reported previously [19]. So far, numerous transgenic animal and mammalian models have been established as in vivo experiments to elucidate the cell death mechanism and evaluate neuroprotective agents in the retina [20,21,22,23,24,25,26,27,28]. Using these animal models, the effects of some neuroprotective agents against RGC cell death have been investigated, including the pyruvate dehydrogenase kinase inhibitor [20], kynurenic acid [21], simvastatin [22], valproate [23], pasireotide [24], prothymosin alpha [25], and the Jun N-terminal kinase inhibitors [26,27,28]. However, these animal studies significantly differ from the situation in which human RGCs are exposed to ischemia–reperfusion. In vitro, RGCs have been isolated and cultured from a rat retina [29,30]. Nevertheless, RGC isolation and culture from a rat retina are unstable and may not be suitable for a cell death analysis because they have short neurites but not long axons. Further, the differences in the effects and safety of various agents between human and animal cells are a serious problem. In these respects, this model, using human-derived cells, is considered more clinically relevant than the in vivo and in vitro experiments using these experimental animals. On the other hand, several models have been created to create RGCs using human iPSCs [16,17]. These models are superior to our model in terms of the purity of RGCs. However, there are no reports of ischemia–reperfusion in these models, and this model is simple to evaluate whether the RGCs are alive or dead by looking at the number of axons extending radially from the OV.

**Figure 5:**
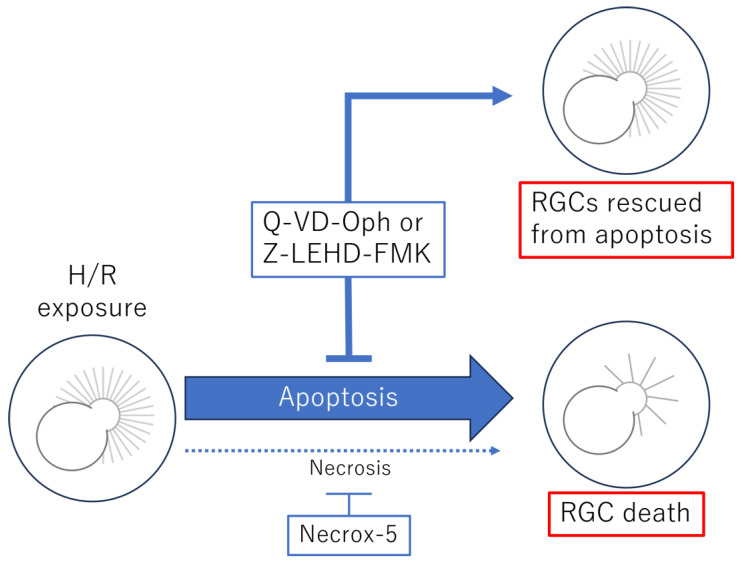
Schema of the axons of retinal ganglion cells (RGCs) before and after hypoxia–reoxygenation (H/R). The reduction in the number of RGC axons after H/R was rescued by the addition of Q-VD-Oph or Z-LEHD-FMK, which prevent apoptosis. On the other hand, Necrox-5, a necrosis inhibitor, could not prevent axons from dying.

We initially expected necrosis-dominant RGC death, as oxidative stress plays an important role in RGC death caused by I/R injury [3,31]. Surprisingly, in our H/R model of human RGCs, only the pan-caspase inhibitor Q-VD-Oph relieved RGC death. However, the necrosis inhibitor Necrox-5, which reduces intracellular calcium concentration and selectively blocks oxidative-stress-induced necrotic cell death, showed minimal protective effects against RGC death, demonstrating that human RGC death in our model was mostly through apoptosis (Figure 5).

Brain ischemia in rats and mice has been primarily used as a model for neural ischemia–reperfusion. Various forms of cell death have been reported in these models, including apoptosis [32], necroptosis [33], ferroptosis [34], and autophagy [35]. In an infant rat I/R model, apoptosis-dominant cell death in the cerebrum was observed, which was achieved by banding the middle cerebral artery. In this model, apoptosis gradually shifts to necrosis as the infant rat grows older, indicating that immature neural cells easily undergo apoptosis after I/R injury [36]. Our established hiPSC-derived RGCs were exposed to H/R only 27 days after the initiation of their development; therefore, an obvious predominance of apoptosis may be observed.

In an in vivo ischemia–reperfusion injury model, several types of death emerge, in which necroptosis appears first in RGCs, followed by apoptosis and ferroptosis [37]. Therefore, blocking the apoptotic pathway alone is insufficient to prevent RGC death because necroptosis and ferroptosis occur in later stages, and the RGCs eventually die. Thus, it is difficult to rescue RGCs unless all the pathways are inhibited simultaneously. However, in our study, we could rescue RGCs from death by suppressing apoptosis alone. 

The main limitation of our H/R system for inducing human RGC apoptosis is as follows: the human RGCs in our experiment were heterogeneous because they were part of the attached retinal primordium; apoptotic cells were found among RGCs as well as retinal primordial cells [15]. Thus, H/R experiments after isolating human RGCs from the retinal primordium may be ideal; however, the typical axons of RGCs cannot be maintained and are destroyed after digestion and isolation. The axonal phenotype is only observed when RGCs exist in the peripheral region of the attached retinal primordium, which facilitates an evaluation of the effectiveness of apoptosis inhibitors based on morphological changes.

## 5. Conclusions

In summary, compared with previous models, our model is unique as it focuses on RGCs, which are most vulnerable in the retina and are apoptosis-driven. Our model is the first I/R model of RGCs created using hiPSCs and will provide even more clinically relevant results than experiments conducted using animal models. Our model is also more visually straightforward in quantifying cell death. Our established H/R model may be useful for investigating the in vitro molecular biology of apoptosis, the most common and initial step of cell death in the retina and the nerves, evaluating cell death through the apoptosis pathway, and assessing the effectiveness of apoptosis inhibitors.

## Data Availability

The data presented in this study are available on request from the corresponding author.

## Supplementary Materials

The following supporting information can be downloaded at https://www.mdpi.com/article/10.3390/cells13020130/s1: Figure S1: Staurosporine-treated RGCs and H_2_O_2_-treated RGCs showed a remarkable increase in apoptotic and necrotic cells compared to those in normoxic cells.

## Abbreviations

RGC	Retinal ganglion cell
iPSC	induced pluripotent stem cell
hPSC	Human pluripotent stem cell
ESC	Embryonic stem cell
I/R	Ischemia/reperfusion
H/R	Hypoxia–reoxygenation
MEF	Mouse embryonic fibroblasts
bFGF	Basic fibroblast growth factor
RDM	Retinal differentiation medium
D0, D1, D2	Day0, Day1, Day2
FBS	Fetal bovine serum
CHIR99021	Glycogen synthase kinase 3β inhibitor
SAG	Smoothened agonist
RMM	Retinal maturation medium
BDNF	Brain-derived neurotrophic factor
NF	Neurobasal medium
Q-VD-Oph	Pan-caspase inhibitor
Necrox-5	necrosis inhibitor
Z-LEHD-FMK	Caspase-9 inhibitor
OV	Optic vesicle
