# Supplementary material for: Modeling of Retina and Optic Nerve Ischemia–Reperfusion Injury through Hypoxia–Reoxygenation in Human Induced Pluripotent Stem Cell-Derived Retinal Ganglion Cells
Source: Cells. 2024 Jan 11;13(2):130. doi: 10.3390/cells13020130 (PMC10814087; doi:10.3390/cells13020130)
Supplement: Supplementary file 1 [file cells-13-00130-s001.zip › cells-2769706-supplementary.pdf]

Supplementary Methods for Figure S1

Immunohisotochemistry.

Biological staining was performed with CellEvent™ Caspase-3/7 Green Detection Reagent and SYTOX™ AADvanced™ Dead Cell Stain. The culture medium in the plate was removed once, culture medium with CellEvent™ Caspase-3/7 Green Detection Reagent was added, and incubated in the dark for 20 minutes, followed by incubation with culture medium containing SYTOX™ AADvanced™ Dead Cell Stain for another 5 minutes in the dark for final observation.

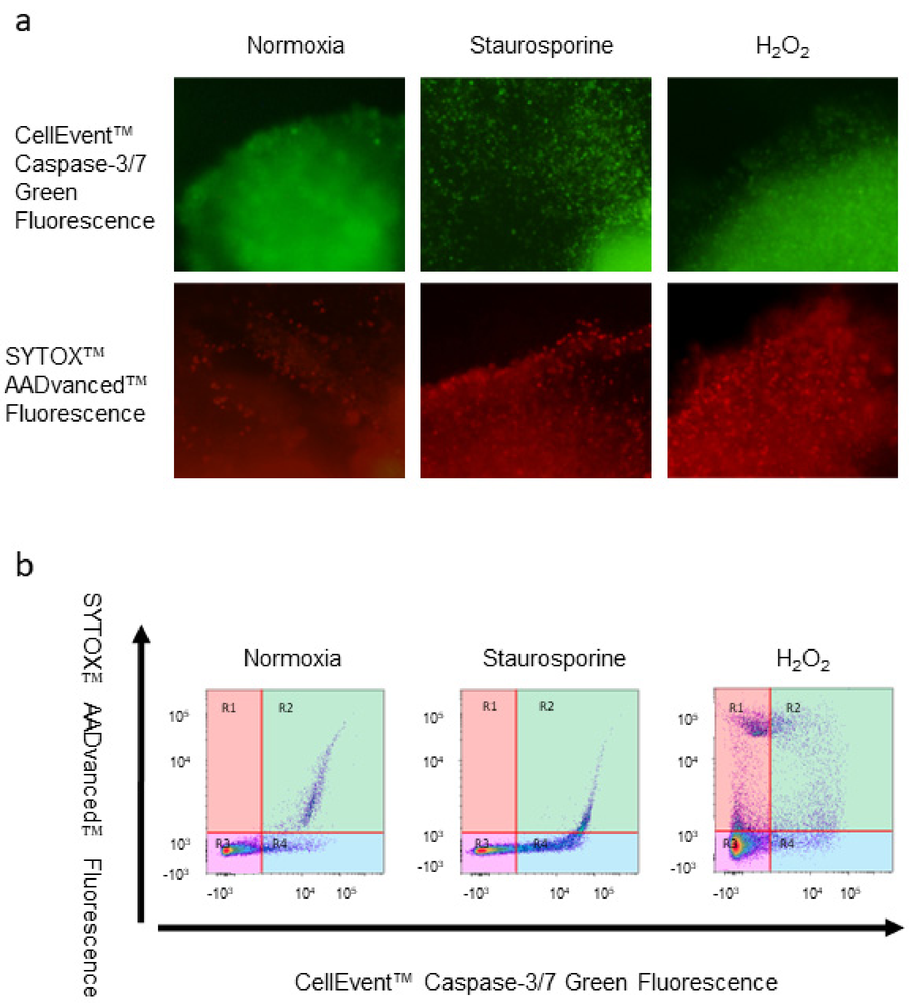

Figure S1.

Staurosporine-treated RGCs and H_2O_2-treated RGCs showed a remarkable increase in apoptotic and necrotic cells compared to those in normoxic cells.

(a) Immunohistochemistry of RGCs in normal air (normoxia), RGCs with Staurosporine, and RGCs with H_2O_2. Images were obtained 3 hours after drug administration. SS induced apoptosis and H_2O_2 induced necrosis. (b) Flow cytometry results under normoxia, Staurosporine and H_2O_2 conditions. Treatment with Staurosporine, an apoptosis inducer, increased the number of early apoptotic cells in the R4 region. When cells were treated with H_2O_2, a necrosis inducer, the number of cells in the R1 region increased, indicating the presence of necrotic cells.

RGC, retinal ganglion cell.
